# Hydrology Affects Environmental and Spatial Structuring of Microalgal Metacommunities in Tropical Pacific Coast Wetlands

**DOI:** 10.1371/journal.pone.0149505

**Published:** 2016-02-22

**Authors:** Carmen Rojo, Francesc Mesquita-Joanes, Juan S. Monrós, Javier Armengol, Mahmood Sasa, Fabián Bonilla, Ricardo Rueda, José Benavent-Corai, Rubén Piculo, M. Matilde Segura

**Affiliations:** 1 Cavanilles Institute for Biodiversity and Evolutionary Biology, Universidad de Valencia, Paterna, Spain; 2 Departamento de Biología, Facultad de Ciencia y Tecnología, Universidad Nacional Autónoma de Nicaragua, León, Nicaragua; 3 Instituto Clodomiro Picado, Facultad de Microbiología, Universidad de Costa Rica, San José, Costa Rica; University of Aveiro, PORTUGAL

## Abstract

The alternating climate between wet and dry periods has important effects on the hydrology and therefore on niche-based processes of water bodies in tropical areas. Additionally, assemblages of microorganism can show spatial patterns, in the form of a distance decay relationship due to their size or life form. We aimed to test spatial and environmental effects, modulated by a seasonal flooding climatic pattern, on the distribution of microalgae in 30 wetlands of a tropical dry forest region: the Pacific coast of Costa Rica and Nicaragua. Three surveys were conducted corresponding to the beginning, the highest peak, and the end of the hydrological year during the wet season, and species abundance and composition of planktonic and benthic microalgae was determined. Variation partitioning analysis (as explained by spatial distance or environmental factors) was applied to each seasonal dataset by means of partial redundancy analysis. Our results show that microalgal assemblages were structured by spatial and environmental factors depending on the hydrological period of the year. At the onset of hydroperiod and during flooding, neutral effects dominated community dynamics, but niche-based local effects resulted in more structured algal communities at the final periods of desiccating water bodies. Results suggest that climate-mediated effects on hydrology can influence the relative role of spatial and environmental factors on metacommunities of microalgae. Such variability needs to be accounted in order to describe accurately community dynamics in tropical coastal wetlands.

## Introduction

According to metacommunity theory, community assembly processes can follow a neutral model or be explained by differences among species related mainly to the adaptation to local environmental factors and to differential dispersal abilities [1–2].

In that sense, geographical spatial extent of observation would partly explain biodiversity distribution [3–4]. One well stablished idea is that aquatic communities may be more strongly structured at large spatial extents by dispersal-related processes, evolution, and historical events [5–6] than by local environmental factors and biotic interactions.

If attention is focused on the types of organisms or their traits, then body size is found to be inversely related to dispersal because decreasing size increases the dispersal rate of passively dispersing species [7–8]. That is, communities of smaller species are more controlled by the local environment while those of larger species are more strongly affected by space [1,9]. In fact, variance in the distribution of very small aquatic microorganisms such as diatoms can be explained by both local conditions and geographical distance [10–13]. Moreover, structure of microalgal metacommunities depends not only on different fractions of body sizes but also on life forms [6,13–14]. However, there are still few studies of microalgal metacommunities and even less on the different responses that depend on functional groups within microalgae [14].

An overview of the studies dealing with spatial and environmental effects on microalgal assemblages reveals a bias towards temperate areas. In those regions, the thermal regime follows a seasonal pattern that is considered very important for the structuring processes (the classic view by [15]). However, most studies consist of snapshot surveys, with some exceptions such as that of [11], who emphasize intra-interannual effects on diatom structuring and encourage other authors to consider dynamics in their studies of metacommunities. Therefore, there is a major gap in the analysis of spatiotemporal structure of aquatic communities from climatic regions with severe intra-annual hydrological changes, such as Mediterranean or tropical dry forest regions [6,16–18]. Moreover, in the Pacific Coast of Central America there are losses of original area covered by seasonal wetlands due to severe anthropic transformations [19–20]. Climate and anthropic changes are reducing aquatic systems in one of the most vulnerable tropical ecosystem, the tropical dry forest whose biodiversity is highly dependent on their wetlands [21]. However, and despite their important role, there is currently little information available on the conservation status of wetlands in this region [22–23]. Lack of information on tropical plankton communities and their structuring processes in wetlands and ponds [24] is a major drawback in view of the effects that the ongoing climate change is having on the sensitive algae to warmer scenarios and on the hydrological functioning of tropical dry forest [25–26].

In agreement with both the paradigms mentioned above on metacommunity structuring and recent results, our hypothesis states that climatic factors, such as the precipitation regime, can modify the balance between niche-based and neutral processes. Periods of rain coupling with flooding have the opposite effect of periods of dryness within isolated and smaller water bodies in planktonic foodwebs from semi-arid Mediterranean regions [27,28] and in areas of tropical wet and dry climate [16,29].

In the wake of studies on the relationship between large spatial extents and their likely regional heterogeneity, and the primary mechanisms that structure metacommunities [6,13,30], we aimed at understanding the added effects of wet-dry dynamic hydrology on highly dispersive, small body sized organisms such as microalgae. We expect variations in niche and neutral effects when the climate alternates between rain and dry periods. Rain periods imply flooded areas, more connectivity between water bodies and strong physical local perturbations. On the contrary, dry periods favour isolated areas that have reduced water bodies, which implies reduced connectivity but changing local conditions structuring aquatic communities.

In summary, we hypothesize that i) environmental and spatial factors may have variable roles in structuring microalgal metacommunities depending on body size of the algal groups; ii) at large spatial extents, distribution of phytoplankton may significantly differ between regions; and iii) during desiccation, local processes become more relevant.

## Materials and Methods

### Study areas

**Fig 1:**
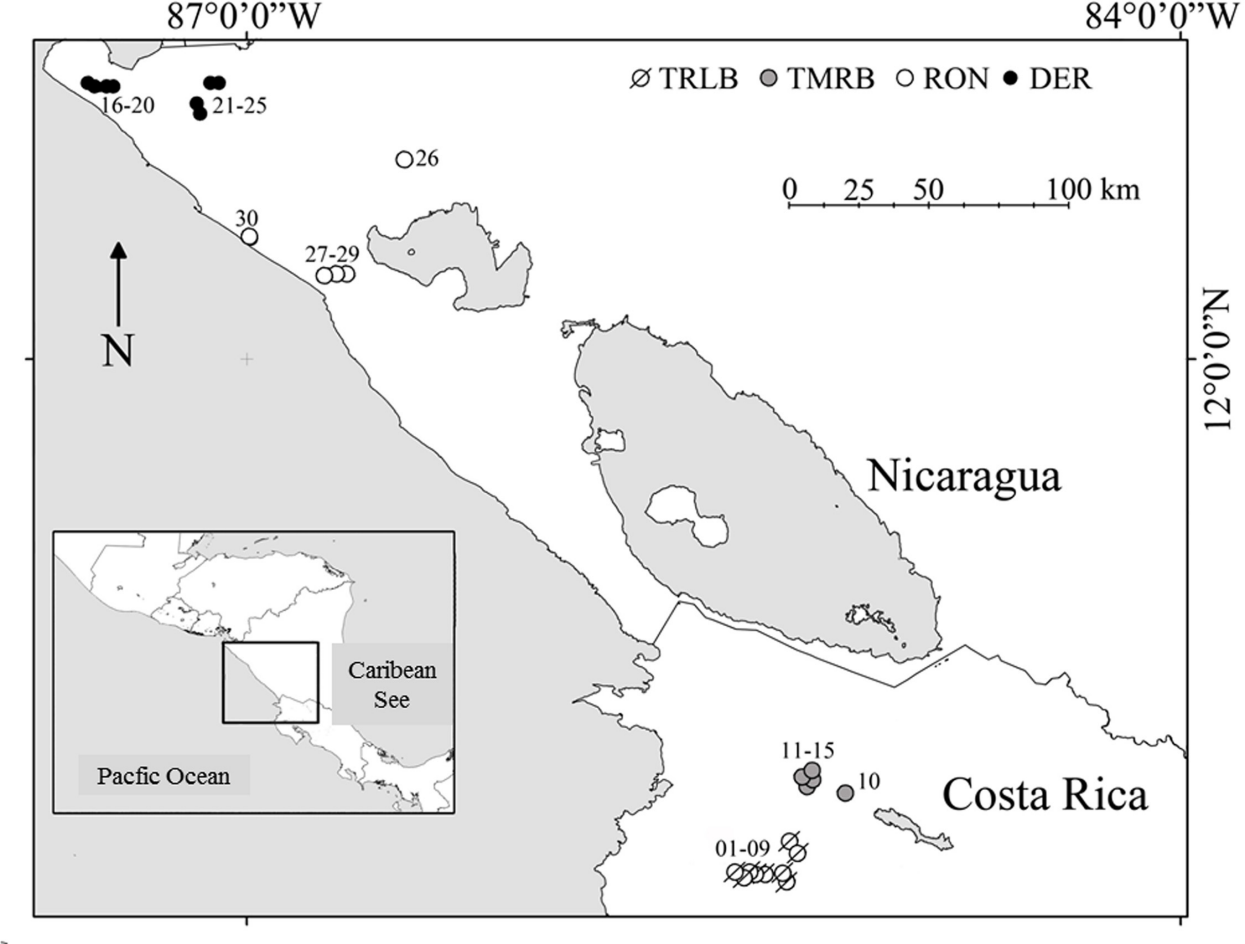
Map of the study area (Costa Rica and Nicaragua Pacific coasts) indicating sampled aquatic ecosystems. Water bodies numbered as in S1 Table. Reprinted from [31] under a CC BY 4.0 license (http://creativecommons.org/licenses/by/4.0/us/), with permission from *Asociación Ibérica de Limnología* (Limnetica), original copyright 2014.

Microalgae were collected from 30 water bodies in a region along the Pacific tropical coast of Costa Rica and Nicaragua (Fig 1). These ponds or lagoons belong to four regions: (1) the lower section of the Tempisque River Lower Basin (TRLB) in the Palo Verde National Park and surrounding areas, (2) slopes of the volcanoes Miravalles and Tenorio in the Tempisque River Middle Basin (TRMB), (3) estuarine wetlands of the Delta del Estero Real (DER) with tidal influences from the Golfo de Fonseca and (4) ponds in the western Region of Nicaragua (RON) located within 50 km southwest of the city of León.

These wetlands were chosen following three criteria: a) to focus on a climatic region that has extreme hydrological dynamics, with periods alternating between dry and flooded conditions; b) to cover a large spatial extent within the same tropical dry forest climate type; and c) to sample a set of water bodies with varied typology, connectivity, and nutrient concentrations. The selected water bodies are distributed from 12° 53' 22.6'' N at the “Estero Real” estuary in Nicaragua to the “Nicaragua” lagoon at 10° 19' 42.9'' N in Costa Rica (Fig 1 and S1 Table). These sites were sampled three times: in June 2010, few weeks after the tropical rain period had begun, September 2010 when the flooding was highest and January 2011 at the end of the rainy season (more information on precipitations and details of sampling sites can be found in [31]). Hereafter, these three hydrological periods will be referred to as Infilling, Flooded and Desiccation periods, respectively.

Although water body features can display a high degree of spatial autocorrelation complicating the task of disentangling the effects of the environment from spatial factors [12], a previous analysis on these water bodies revealed a reduced limnological similarity between the sites closest to each other [31]. Some morphological, physico-chemical and biological information for each water body is listed in S1 Table and more details are given in [31].

### Ethics statement

We obtained an authorization from the *Ministerio de Ambiente*, *Energia y Telecomunicaciones de Costa Rica* to take samples from the protected areas (Tempisque River basin, Palo Verde National Park). In order to obtain samples in ponds located in private properties we obtained verbal permissions from the owners. Our field study was focused on plankton and did not involve endangered or protected species.

### Field sampling and laboratory analysis of microalgae

Microalgal samples were collected just below the surface of the water with a jar attached to an extendable arm, avoiding disturbing the sediment as much as possible. Because of the shallow character of the water bodies and the common presence of macrophytes, samples mostly contained phytoplankton but also populations that are considered benthonic or tychoplankton. We included these populations in our analysis because they can be an important fraction of the primary producers in shallow freshwater [32], and they can be especially relevant in shallow places subjected to perturbation and drought periods [27]. Collected samples were fixed immediately with acid Lugol’s iodine solution in the field.

To study microalgae, populations in aliquots from the fixed material were observed, measured and counted with an inverted microscope. The numbers of counts were designed to obtain 9% of the total density error [33], and the biomass of the population was calculated according to geometric equations and expressed as the individual volume of the species multiplied by the abundance of individuals (biovolume, mm^3^ L^-1^). Monographs and specific reviews were consulted to classify the populations of species and discriminate them as benthonic or planktonic populations. After building an initial species composition matrix we excluded from further analyses those species with a contribution < 5% to the total community biomass in any sample, following [34]. For some analyses, the obtained dataset was split in two matrices: one that only included phytoplanktonic populations and another that only included the tychoplanktonic (benthic) microalgae.

### Environmental and geographical variables

The 24 variables used can be seen in S2 Table. The maximum lake depth was measured in the middle of the water body during the flooded season sampling campaign. Studied sites were georeferenced, mapped and measured (area and perimeter) over GoogleEarth® images with ArcGIS 10.0 [35]. Analyses of the surface-water temperature, conductivity (EC), and pH were conducted using a Hanna® pH/EC meter HI 98130, oxygen concentration (mg L^-1^) with the Winkler method and a Snell tube served to estimate water transparency [36]. Samples for nutrient analysis were collected in acid-washed 100 mL HDPE bottles, stored in ice boxes, and frozen some hours later. Water for soluble nutrients was filtered through GF/F Whatman filters in the field. An unfiltered sample of water was collected in a 250 mL bottle and stored at -20°C for anion analyses. Another 100 mL sample of water was fixed with nitric acid for subsequent cation analyses. The chemical analyses included dissolved nutrients (nitrate, nitrite, ammonia and phosphate), major anions (chloride, sulphate and alkalinity) and cations (Ca^+2^, Mg^+2^, K^+^ and Na^+^). Nutrient concentrations and the dissolved solids were analysed following Standard Methods [37]. For the chlorophyll-*a* analyses, water was filtered using a GF/F Whatman filter and analysed spectrophotometrically after extraction with acetone following [38]. The macrophyte coverage, as percentage of cover area, was visually estimated by walking several transects. Further details on sampling and environmental data acquisition methods are described in [31].

### Statistical analyses: datasets and variance partitioning

The abundances (biovolumes) of the three microalgal matrices (total microalgae, the phytoplankton fraction and the benthic fraction) were Hellinger-transformed to equilibrate the influence of both the common and rare species [39–40]. Because the posterior analyses should be conducted on detrended data [40–41], we first tested if these community matrices contained a linear gradient by applying a redundancy analysis (RDA) with community composition matrices as the response variables and geographic coordinates as the explanatory variables. If a linear trend was detected, it was removed for each species (i.e. matrix columns) separately and a detrended matrix was constructed with the residuals [42].

The spatial structure was summarised at multiple scales with a principal coordinate neighbour matrix (PCNM; [41–43]). PCNMs with higher eigenvalues represented broad-scale variation, whereas PCNMs with lower eigenvalues corresponded to fine-scale variation [43]. To identify the spatial factors (eigenvectors and PCNMs) that explained a significant amount of the variation in the detrended community composition matrices, we applied a forward selection stepwise procedure with two stopping criteria [44]. However, this forward selection was only applied on PCNMs that explained or almost explained above 5% of the community variance to reduce the spurious or irrelevant variables prior to the forward procedure. The criterion of 5% does not require a statistical test because such significance does not reflect the importance of variance but the strength of confidence in making the inference [45]. The spatial scale or distance associated with each selected eigenvector was quantified with a graphical representation of the spatial distribution of wetlands (abscissae—x-coordinates; ordinates—y-coordinates) in which the symbol size and colour of each wetland were dependent on the magnitude and sign of the principal coordinates score, respectively [46–47]. The first eigenvectors (i.e. D1-D3), which are associated with large eigenvalues, corresponded to variations at a broad distance, whereas the eigenvectors associated with small eigenvalues (i.e. D8-D10) represented variations between neighbouring lakes [10].

The environmental structure was summarised in a matrix with the scores of a principal component analysis (PCA) applied to the environmental variables; with this method, we reduced the number of variables based on the number of cases and also reduced the covariate variables. Because the environmental factors were measured in different units, we standardised the variables to control for differences in variance and better detect the unimodal relationships between the community composition and environment [48]. To easily obtain interpretable eigenvectors and prevent the loss of relevant environmental information, we performed a PCA for pre-defined groups of physico-chemical related variables (S2 Table). The lack of collinearity among the eigenvectors resulting from the different PCAs was verified. To identify the environmental factors (PCAs) that explained a significant amount of the variation in the detrended community composition matrices, we applied a Redundancy Analysis (RDA) with forward selection stepwise procedure with double stopping criteria [44]. Similar to the spatial vectors, only the PCAs explaining approximately 5% of the community variance were considered in the forward selection procedure. The same procedure was followed for each set of environmental features during the three hydrological periods (infilling, flooded and desiccation seasons) to produce three environmental matrices with PCA values available for an analysis of the partition of variance.

To verify that each dataset component matched both the three hydrological periods and the three biotic groups (all microalgae, phytoplankton and benthic populations), we tested if the community composition was explained by the environmental or/and spatial factors. Variance partitioning [40,47,49] was performed in RDA to calculate the proportion of variance explained by the environment independent of space [E|S], space independent of the environment [S|E] and shared by the environment and space [E∩S]. Monte Carlo permutation tests (999 runs) were used to evaluate the statistical significance of the variance partitioning [49,50]. The variation explained by each variable group (spatial or environmental) was estimated using adjusted R^2^, which provided unbiased estimates of the explained variation [49]. The use of adjusted R^2^ values often decreases the percentage of variation that is explained and results in a considerable amount of unexplained variation [9].

The PCNM and forward selection analyses were performed with the Matlab language of technical computing (MATLAB R2011b, Atlanta, Georgia); the threshold distance in the PCNM analyses was calculated with the Fathom Toolbox [51], and a redundancy analysis for variance partitioning was conducted with the function from [49].

## Results

We have determined 293 species of microalgae, 200 of which are planktonic, and the rest are considered as mostly associated to benthic habitats (see S3 Table). This classification was related to the average body size of the individuals: most species belonging to the phytoplankton had a major axis mean length (± standard deviation) of 36.8 ± 45.3 μm, while benthonic species measured 108.4 ± 107.2 μm, these values being significantly different (Mann-Whitney test, p < 0.0001). Because taxa having less than 5% of total biomass in each sample were removed, the final matrix with all sampled water bodies over the three hydrological periods contained 95 taxa of microalgae.

When using geographic distances between all 30 sites, with a maximum distance of approximately 370 Km (Fig 1), the number of selected PCNMs was 16. The 24 environmental variables measured were reduced according to a series of PCAs for groups of related variables, resulting 13 selected PCAs for each hydrological period. Environmental variables with higher weight in selected PCs were (see S2 Table): lake morphology (area, perimeter and depth) and altitude, dissolved O_2_ concentration, water temperature and transparency, total dissolved solids (TDS), which is directly related to conductivity and ionic concentrations, and HCO_3_ and nutrients (inorganic nitrogen compounds and soluble phosphorous concentrations), in addition to macrophyte cover.

**Fig 2:**
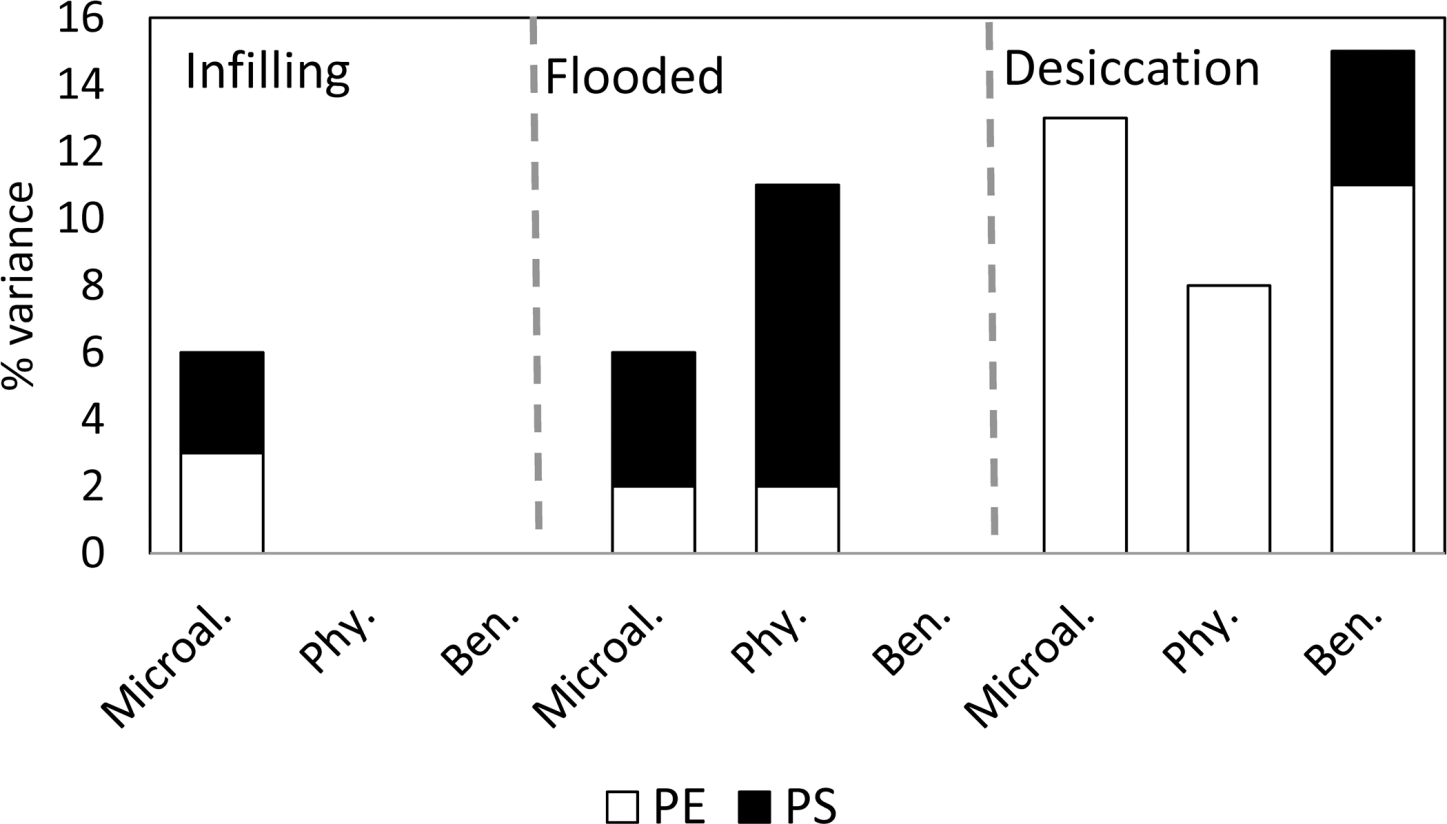
Adjusted variance (% adjR^2^) explained by pure environmental (PE) or pure spatial (PS) factors on the structure of the selected matrices of all microalgae (Microal.), the phytoplankton (Phy.) and benthic (Ben.) fractions for each hydrological period (infilling, flooded, desiccation).

The variance of microalgal distribution was significantly explained by both the pure environmental (PE) and the pure spatial (PS) fractions (Fig 2 and Table 1). These fractions of variance were different depending on the hydrological period: 3% of the species variance was explained by each PE and PS in the infilling period; during the period of flooding, spatial variables explained twice as much variance (4%) as environmental factors, and the opposite was observed during the desiccation period when 13% of the variance was attributed only to PE variables (Table 1). The environmental factors that had significant effects on microalgal distribution were PCs related to TDS during the infilling periods, to oxygen content during the flooded periods and to TDS, dissolved oxygen, macrophyte cover and water transparency at the dry season (see S2 Table).

**Table 1:** Variation partitioning results of a series of (partial) RDAs (each hydrological period was analysed separately). Three matrices of microalgae biomass population were used: all microalgae in the plankton, strictly planktonic populations (phytoplankton) and benthic microalgae (phytobenthos).

GROUP	Fraction	Infilling			Flooded			Desiccation		
		var%	p	Var.	var%	p	Var.	var%	p	Var.
*All microalgae*	E	6	0.01	Env8	6	0.03	Env5	9	0.01	Env8
		-			-			7	0.01	Env5
		-			-			7	0.01	Env1
		-			-			6	0.01	Env6
	S	6	0.01	D4	8	0.01	D3	6	0.02	D1
		-			-			5	0.04	D2
	E|S	3	0.01		2	0.03		13	0.01	
	S|E	3	0.01		4	0.01		-	n.s.	
*Phytoplankton*	E	-	n.s.		7	0.02	Env5	7	0.01	Env13
		-			-			6	0.03	Env8
		-			-			6	0.04	Env1
	S	6	0.04	D4	10	0.01	D3	6	0.02	D2
	E|S	-	n.s.		2	0.03		8	0.01	
	S|E	-	n.s.		9	0.01		-	n.s.	
*Phytobenthos*	E	8	0.01	Env5	-	n.s.		10	0.01	Env8
		7	0.04	Env3	-			6	*0*.*07*	*Env11*
	S	-	n.s.		-	n.s.		7	0.02	D10
	E|S	-	n.s.		-	n.s.		11	0.01	
	S|E	-	n.s.		-	n.s.		4	0.01	

Fraction of variance explained by environment (E) or space (S). Explanatory environmental (Env#) and spatial (D) variables (Var.) used in the RDAs are explained in the text and S2 Table. The amount of explained variability is var% and *p* represents the probability values. Variance explained by pure environmental (E|S) or pure spatial (S|E) variables is expressed as the adjusted R^2^ cumulative value. Italic fonts are used when E|S or S|E were significant but the explanatory variable was not statistically significant (p > 0.05).

**Fig 3:**
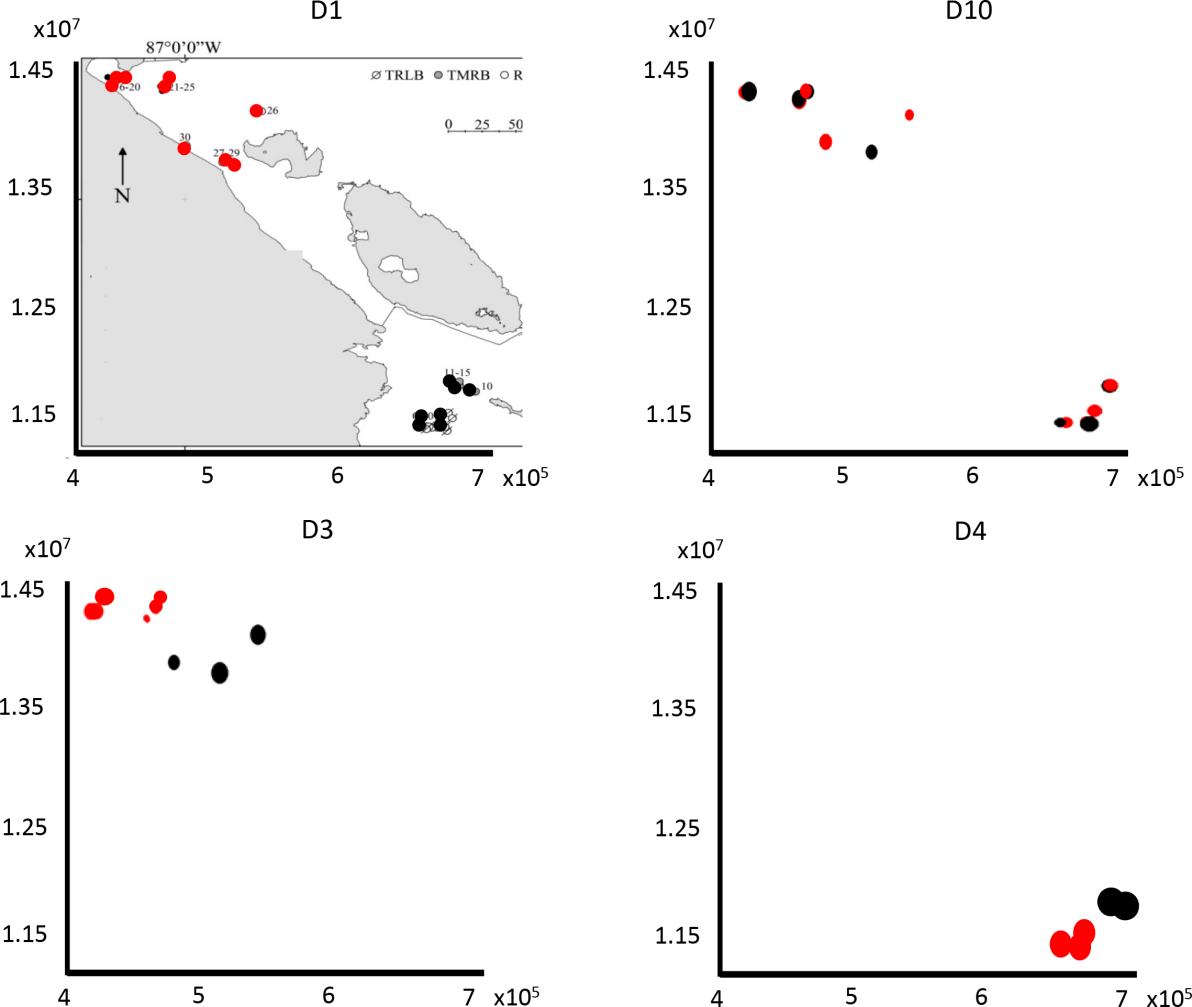
Variation in the site scores of each selected PCNM variable (D1, D3, D4 and D10) along north (y-axis) and east (x-axis) coordinates in the Pacific coast of Nicaragua and Costa Rica. An overlaid map of the region (as in Fig 1) is included in the first graph. The larger black or red dots: the higher positive or negative absolute values for a site respectively.

The spatial components (PCNMs) significantly explaining community structure of microalgal biomass were also different for each hydrological period (Table 1): D4 was significant during the infilling period, separating the water bodies in Tempisque River Lower Basin (TRLB) from those in Tempisque River Middle Basin (TRMB) in Costa Rica (Fig 3); D3 was important in the flooded period, and segregated sites in the western Region of Nicaragua (RON) from Delta del Estero Real (DER) wetlands in the Nicaragua set (Fig 3); and D1-D2, which separated the most distant sets, were the most important PCNMs for the desiccation period (Fig 3).

When only phytoplankton species were used in the analyses, the results slightly changed: in the infilling period, D4 was the only variable selected; in the flooded period, the phytoplankton distributions showed similar structuring factors and fractions of variance explained as those resulting from analysing the global microalgae set. In the desiccation period, PCs related to PO_4_ concentration, TDS, and macrophyte cover and D2 (broad distances) were the environmental and spatial variables selected. However, only PE factors were significant in this analysis (Fig 2). A very different pattern to that of phytoplankton occurred when only the benthic algae were considered (Table 1). During the infilling period, PCs related to oxygen content and depth significantly explained part of the variation in the benthic species distribution, however the PE fraction was not statistically significant; in flooded periods, no variables were selected and during the desiccation periods, only a TDS-related PC was selected and PE explained up to 11% of the variance, while the PS fraction explained 4% of the variance in the species data (Fig 2); in this case being the principal coordinate D10 which was influential, emphasizing differences between close water bodies (Fig 3).

## Discussion

### Microalgal distribution in tropical Pacific coastal wetlands according to niche-based models

Despite the fact that microalgae are considered ubiquitously distributed [52], because of their small body size and high passive dispersal capacity [8], their structuration along the studied water bodies were shaped by both environmental factors and geographical location. This fact is in accordance to previous studies on these aquatic organisms in different landscapes (i.e. [3,5,10]) and suggests that the microalgal distribution pattern fits well to a combination of both neutral and niche-based models, as expected [3,12,30]. Despite the low explained variance, our results were considered as having an ecologically meaningful basis as results obtained in other aquatic communities (e.g. [3,10]). Indeed, our study provides additional information on the metacommunities of small organism as demanded by [53].

Geographic distances alone never explained a significant portion of microalgal (or its phytoplankton fraction) assemblages, so their distributions were not organized in agreement with the neutral model but environmental factors were always involved. Space, when significant, explained more variance of microalgal distribution than environmental factors, the latter never including resources (nutrients), but only variables such as oxygen content, total dissolved solids and water ionic composition. Therefore, microalgal spatial distribution seems to be based on a system of patches that filter the populations through trait selection mechanisms instead of competition for resources. In this sense, a mass effects perspective appears to be more applicable than models of species-sorting based on competition mechanisms or patch dynamics based in sites that are limnologically similar [2].

On the other hand, particularly during the drying phase, the importance of environmental control was stronger than that of pure spatial influences. And at this period, for the phytoplankton functional group, the perspective of species sorting, in which nutrients (PO_4_ concentrations) or light availability (water transparency, macrophyte cover) were the controlling environmental factors appeared as the most fitted framework [2,54]. This pattern of phytoplankton metacommunity distribution supports the relevance that phosphorus concentration, transparency and macrophytes have in structuring phytoplankton in tropical wetlands. Specifically, among the studied variables, these three selected factors are involved in the alternative states of temperate shallow lakes [55] and such regime shifts are now being tested for tropical and subtropical ecosystems [56–58]. This recent interest is related to the hypothesis about the different role of macrophytes (i.e. refuge of organisms, underwater light climate) comparing shallow lakes of different areas to those of temperate Europe [56, 59].

### Large spatial extents uncover the relative relevance of distance-structuring of microalgae

A large spatial extent implies increasing limitations to organisms’ dispersal but also an increase in environmental heterogeneity. Consequently, it is difficult to evaluate the independent contribution of each group of factors structuring metacommunities (e.g. [60]). In the geographic area here considered, the microalgal assemblages were affected by different spatial scale distance limitations, thus meaning that part of the metacommunity structure was explained by distance decay [3,5]. Therefore, focusing on such large extent allowed disentangle the relative relevance of long distance in structuring microalgal communities as compared to the effects on water bodies located at landscapes of smaller extent. We can express that there exist a metacommunity of microalgae at the coastal Pacific region and that its structure is affected both by limited dispersal and high environmental heterogeneity [5].

Our results highlighted that microalgae were structured within two latitudinally-separated regions along the Pacific coast (Costa Rica and Nicaragua) that emerged when broad extent spatial variables (D1 or D2) were selected as main factors. In such case, environmental heterogeneity was included in their different latitudinal location, as it was shown by [61]. Such a structuration was possible when the dry period prevented environmental homogenisation and organismal dispersal [62]. Moreover, our study suggested that including large distances in the analyses enabled to disentangle community heterogeneity between basins. For example, this approach suggested that the microalgal community living in the lower area of Tempisque river (at sea level) which is filled with the first rains of the hydrological year, was different from that inhabiting in waters of the Tempisque river middle basin, located at 340 m a.s.l. and 45 km away, which were mainly artificially-isolated ponds [31]. Similar segregation occurred between two studied Nicaraguan basins, namely those of the permanent RON area and the water bodies close to the temporary environments of Estero Real estuary, located 100 km apart.

Furthermore, our results indicated that the factors controlling the assembly of microalgae impact with different intensities on the two functional groups that comprise these microalgae. In our study, and according to the method proposed by [6], we were able to compare the responses of small body-sized species (planktonic) with larger-sized species (benthic algae). Even within the group of small and ubiquitous organisms differences were found in their metacommunity patterns of organization, in accordance with previous studies, for instance on small diatoms groups [14]. The benthic fraction was structured mainly by local pure environmental factors solely during the desiccation period when shallow water bodies were isolated from each other; its spatial structure was marked by differences between each water body and its neighbour. Therefore, metacommunity structuration was sensible to season, landscape type, geographical situation and functional group, point of view defended by [63].

### Climatic regime: a major factor to be considered in metacommunity analysis

When discussing different factors involved in microalgal metacommunity organization, it is evident that hydrology is related with many limnological and spatial factors. When flooding processes occur in areas along the Pacific tropical coast of C. Rica and Nicaragua, connectivity increases, and the heterogeneity of the wetland area decreases. Flooding promoted spatial de-structuring and limnological homogenization, as already described for river floodplain systems (RFSs) in tropical areas by [64]. Seasonal flooding allowed overall dispersal of a wide range of species, as also observed in fish metacommunities by [65]. On the contrary, the drying periods with higher isolation and heterogeneity of local factors are linked to a greater beta diversity of assemblages, a pattern described in both Mediterranean wetlands and tropical RFSs [27, 64]. Therefore, desiccation periods might enhance assembly distributions that are based more on a species-sorting model, provided by the previous flooding. In our survey, during the desiccation periods when the already isolated water bodies were not completely dry yet, environmental factors explained the highest percentage of variance. Nevertheless, just after infilling, pure spatial factors unexpectedly explained larger amount of variance in microalgal community structure, particularly that of phytoplankton. These contrasting results might be related to a time lag between infilling of isolated water bodies, each with its own propagule bank (fulfilled in previous hydroperiods). After infilling, inoculum from propagule bank causes initial community development independently (with spatial effects related to isolation), but later after heavy rainfall there is a process of homogenisation resulting from large floodings [64–65]. Flooding reduces the effects of local factors on species sorting, yet it takes some time to facilitate dispersal among sites and, therefore, spatial structure originated in the previous period is still present during flooded periods. According to our results, for aquatic communities the annual hydrological sequence would start at the onset of the rainy period, after drought, when no structuring factors of microalgae were detected as a result of initially disturbed rainy period and chaotic dynamics of growing populations. After a time lag when flooded is at its maximum, metacommunity is spatially structured (perhaps due to the combination of previous isolation of developing communities in water bodies and spatially structuring environmental factors [65]). Subsequently, a new time lag during which dispersal and different successional processes occur until reaching the maximum isolation and drought, results in a niche-based metacommunity structure, as described in subtropical lakes in flooding areas [66]. During dry periods, at the end of the hydrological year, the diversity of assemblages mainly depends upon the composition of species transported during the previous flood [64], and the sequence of population substitutions takes place as responses to the localized environmental characteristics and the species-sorting mechanisms.

It is quite evident from our results and those of previous studies (e.g. [65, 67]) that no single snapshot metacommunity analysis can provide a well-established conclusion on the relative roles of niche and neutral processes for a given area and group of organisms, at least when some disturbances or temporal variations exist affecting the assembly of communities. However, understanding the dynamics of environmental factors and connectivity pathways in dispersing organisms may provide clues about general patterns of this ever-changing distribution of biodiversity in water bodies and landscapes worldwide.

## Supporting Information

S1 Table Geographical location of studied water bodies and certain morphological, physic-chemical and biological features. Studied water bodies, their geographical location and range or mean (M) and standard deviation (SD) of some morphological, physic-chemical and biological features. LAT: latitude; LON: longitude; Tran: water transparency using the Snell tube; Cond: conductivity; Macroph: % macrophyte cover.
(PDF)

S2 Table Results of the PCA on 24 environmental variables describing water bodies during the three sampling periods. Factor coordinates of each variable in the selected PC#; Env# indicated the variables selected as environmental data in the matrix for RDA analyses. Variables in bold are those with extreme loadings for each PC.
(PDF)

S3 Table List of microalgae species found in the study of 30 water bodies in tropical dry forest areas of Costa Rica and Nicaragua sampled at three different hydrological periods during 2010 and 2011. Their life form and body size are indicated. Life form: p-plankton, b-benthic; GALD: greatest axial linear dimension.
(PDF)
