# Supplementary material for: Hydrology Affects Environmental and Spatial Structuring of Microalgal Metacommunities in Tropical Pacific Coast Wetlands
Source: PLoS One. 2016 Feb 22;11(2):e0149505. doi: 10.1371/journal.pone.0149505 (PMC4762632; doi:10.1371/journal.pone.0149505)
Supplement: S1 Table — Studied water bodies, their geographical location and range or mean (M) and standard deviation (SD) of some morphological, physic-chemical and biological features. LAT: latitude; LON: longitude; Tran: water transparency using the Snell tube; Cond: conductivity; Macroph: % macrophyte cover. (PDF) [file pone.0149505.s001.pdf]

Code	Wetland name	LAT	LONG	Area	Tran.	O_2	Cond. (mS)		HCO_3 (meq)		NO_3(meq)		PO_4(meq)		Macroph.
	Costa Rica Region			(Ha)	(cm)	(mg/L)	M	SD	M	SD	M	SD	M	SD	%
1	Torre1 (Palo Verde)	10º 20' 37.5'' N	85º 20' 23.5'' W	1267	21-25	1-6	0.62	0.16	0.4	0.3	0.03	0.01	0.0035	0.0012	85-90
2	Lazaro (Palo Verde)	10º 20' 45.8'' N	85º 21' 08.9'' W	1267	12-28	2-3	0.23	0.09	0.2	0.1	0.03	0.01	0.0080	0.0098	95
3	Torre2 (Palo Verde)	10º 20' 27.6'' N	85º 20' 37.4'' W	1267	8-50	1-5	0.40	0.28	0.3	0.1	0.02	0.02	0.0036	0.0028	75-100
4	Huerton (Palo Verde)	10º 20' 37.9'' N	85º 21' 48.3'' W	1267	15-18	1-4	0.27	0.23	0.2	0.0	0.03	0.00	0.0038	0.0015	30-70
5	Bocana	10º 20' 55.4'' N	85º 16' 48.8'' W	331	24-35	0-2	0.25	0.12	0.2	0.1	0.03	0.00	0.0020	0.0003	85-100
6	Laguna Nicaragua	10º 19' 42.9'' N	85º 16' 11.1'' W	5370	0-24	1-4	0.85	0.80	0.8	0.0	0.03	0.01	0.0029	0.0008	30-95
7	Laguna Nicaragua	10º 19' 44.4'' N	85º 16' 19.5'' W	5370	23-39	0-2	0.39	0.15	0.3	0.2	0.03	0.02	0.0030	0.0010	50-99
8	Estrella (Tamarindo)	10º 25' 37.8'' N	85º 14' 17.2'' W	1	6-15	3-5	0.09	0.06	0.1	0.0	0.03	0.01	0.0022	0.0007	90-100
9	El Canal (Tamarindo)	10º 26' 23.5'' N	85º 15' 15.8'' W	0	12-27	5-7	0.07	0.01	0.0	0.0	0.03	0.00	0.0052	0.0046	80-100
10	Tenorio	10º 36' 38.8'' N	85º 05' 00.1'' W	3	13-18	7-8	0.07	0.01	0.0	0.0	0.11	0.12	0.0018	0.0000	0-20
11	Altamira	10º 38' 31.6'' N	85º 12' 26.7'' W	1	21-42	0-3	0.22	0.02	0.1	0.1	0.03	0.01	0.0045	0.0027	90-100
12	Sainalosa	10º 39' 56.9'' N	85º 12' 08.0'' W	1	29-49	5-13	0.16	0.02	0.1	0.1	0.03	0.01	0.0018	0.0001	5-120
13	Las Brisas	10º 38' 57.0'' N	85º 11' 57.7'' W	1	7-28	6-8	0.11	0.03	0.1	0.1	0.02	0.01	0.0026	0.0008	1-5
14	Eneas	10º 38' 23.8'' N	85º 12' 22.4'' W	2	24-27	4-5	0.23	0.02	0.1	0.1	0.03	0.00	0.0033	0.0005	60-80
15	La Lagarta	10º 38' 41.7'' N	85º 12' 12.8'' W	2	11-19	1-5	0.20	0.02	0.1	0.1	0.03	0.00	0.0022	0.0006	95-100
	Nicaragua Region														
16	Pueblo Nuevo 1	12º 52' 56.0'' N	87º 29' 01.3'' W	10	25-42	2-3	0.75	0.33	0.5	0.3	0.01	0.01	0.0066	0.0061	30-80
17	Pueblo Nuevo 2	12º 52' 38.6'' N	87º 28' 26.1'' W	2	9-30	1-3	0.96	0.43	0.7	0.4	0.02	0.01	0.0032	0.0014	30-90
18	Playón Caterina 1	12º 52' 57.7'' N	87º 27' 24.7'' W	5	9-18	1-3	2.30	2.23	2.3	0.1	0.02	0.01	0.0045	0.0006	5-95
19	Playón Caterina 2	12º 53' 02.4'' N	87º 26' 32.7'' W	1	20-40	2-4	0.37	0.01	0.2	0.3	0.01	0.00	0.0016	0.0015	5-20
20	Playón Caterina 3	12º 52' 58.3'' N	87º 26' 24.7'' W	0	10-21	3-6	0.44	0.06	0.2	0.3	0.01	0.01	0.0031	0.0003	75-100
21	Tonalá	12º 48' 16.6'' N	87º 08' 49.5'' W	6	14-34	1-4	1.32	1.56	1.4	0.2	0.01	0.01	0.0037	0.0022	40-95
22	Puerto Morazán 1	12º 48' 53.8'' N	87º 09' 08.1'' W	150	20-32	1-4	3.99	2.58	3.3	1.0	0.02	0.01	0.0019	0.0006	80-100
23	Puerto Morazán2	12º 48' 59.8'' N	87º 09' 25.4'' W	150	12-33	3-4	2.25	0.42	1.3	1.3	0.03	0.00	0.0023	0.0004	80-100
24	Estero Real 1	12º 53' 19.3'' N	87º 05' 47.7'' W	15	11-39	5-7	6.28	10.57	8.4	3.0	0.02	0.01	0.0021	0.0008	1-70
25	Estero Real 2	12º 53' 22.6'' N	87º 06' 34.4'' W	8	20-44	5.1	4.13	6.79	5.3	1.3	0.02	0.00	0.0027	0.0015	0-30
26	Dos Montes (Puente)	12º 39' 02.8'' N	86º 29' 53.6'' W	0	10-21	4-6	0.13	0.06	0.1	0.0	0.01	0.01	0.0038	0.0009	0-50
27	San Jorge (Izapa-1)	12º 17' 03.1'' N	86º 43' 22.9'' W	0	7-20	4.9	0.30	0.26	1.8	2.7	0.02	0.01	0.0029	0.0014	30-40
28	San Jorge (Izapa-2)	12º 16' 59.9'' N	86º 43' 29.8'' W	0	7-21	5.3	0.43	0.36	2.0	2.8	0.02	0.01	0.0063	0.0066	25-40
29	Los Corrales	12º 17' 13.4'' N	86º 43' 09.8'' W	1	26-35	5-9	0.38	0.29	0.3	0.1	0.01	0.01	0.0068	0.0090	70-130
30	Palermo	12º 23' 15.5'' N	86º 59' 27.3'' W	10	5-13	3-6	0.19	0.11	0.2	0.1	0.02	0.01	0.0024	0.0007	0-10
