# Supplementary material for: Hydrology Affects Environmental and Spatial Structuring of Microalgal Metacommunities in Tropical Pacific Coast Wetlands
Source: PLoS One. 2016 Feb 22;11(2):e0149505. doi: 10.1371/journal.pone.0149505 (PMC4762632; doi:10.1371/journal.pone.0149505)
Supplement: S2 Table — Factor coordinates of each variable in the selected PC#; Env# indicated the variables selected as environmental data in the matrix for RDA analyses. Variables in bold are those with extreme loadings for each PC. (PDF) [file pone.0149505.s002.pdf]

Infilling period

Variable measured	*Variable used in environmental matrix (Env#)*		
**Macrophytes**	Env1		
	Factor coordenates		
	PC1 (Env2)	PC2 (Env3)	PC3 (Env4)
Max depth	0.19	0.26	0.01
**Mean depth**	0.09	**0.38**	0.10
**Area**	**0.29**	0.17	0.06
**Perimeter**	**0.30**	0.18	0.01
**Altitude**	0.12	0.01	**0.82**
Eigenvalue	2.3	1.6	0.8
% Total variance	45.6	31.5	16.6
Cumulative eigen.	2.3	3.9	4.7
Cumulative%	45.6	77.1	93.7
	PC1 (Env5)	PC2 (Env6)	PC3 (Env7)
**Transparence**	0.01	**0.40**	0.01
**T**	0.29	0.02	**0.60**
pH	0.26	0.19	0.06
**O_2**	**0.41**	0.00	0.12

Flooded period

Variable measured	*Variable used in environmental matrix (Env#)*		
**Macrophytes**	Env1		
	Factor coordenates		
	PC1 (Env2)	PC2 (Env3)	PC3 (Env4)
Max depth	0.16	0.30	0.11
**Mean depth**	0.07	**0.48**	0.07
**Area**	**0.34**	0.07	0.10
**Perimeter**	**0.34**	0.11	0.04
**Altitude**	0.08	0.03	**0.68**
	2.3	1.4	1.0
	45.2	28.5	20.9
	2.3	3.7	4.7
	45.2	73.6	94.5
	PC1 (Env5)	PC2 (Env6)	PC3 (Env7)
**Transparence**	0.02	**0.55**	0.05
**T**	0.16	0.00	**0.52**
pH	0.32	0.07	0.21
**O_2**	**0.43**	0.03	0.01

Desiccation period

Variable measured	*Variable used in environmental matrix (Env#)*		
**Macroph.**	Env1		
	Factor coordenates		
	PC1 (Env2)	PC2 (Env3)	PC3 (Env4)
Max depth	0.19	0.15	0.28
**Mean depth**	0.13	**0.31**	0.02
Area	0.24	0.25	0.01
**Perimeter**	**0.27**	0.21	0.00
Altitude	0.17	0.07	**0.69**
	2.3	1.6	0.7
	46.7	32.3	13.6
	2.3	3.9	4.6
	46.7	79.0	92.6
	PC1 (Env5)	PC2 (Env6)	PC3 (Env7)
**Transpar.**	0.02	**0.57**	0.00
**T**	0.18	0.10	**0.51**
pH	0.31	0.10	0.01
**O_2**	**0.32**	0.13	0.00

Chl a	0.03	0.39	0.21	Chl a	0.07	0.35	0.21	Chl a	0.18	0.11	0.48
Eigenvalue	2.1	1.8	0.5		1.6	1.3	1.1		2.3	1.4	0.7
% Total variance	42.2	35.4	10.4		32.9	25.8	21.1		46.8	28.6	14.2
Cumulative eigen.	2.1	3.9	4.4		1.6	2.9	4.0		2.3	3.8	4.5
Cumulative%	42.2	77.6	88.1		32.9	58.7	79.8		46.8	75.4	89.6
	PC1 (Env8)	PC2 (Env9)	PC3 (Env10)		PC1 (Env8)	PC2 (Env9)	PC3 (Env10)		PC1 (Env8)	PC2 (Env9)	PC3 (Env10)
**TDS**	**0.16**	0.00	0.00	**TDS**	**0.15**	0.02	0.01	**TDS**	**0.17**	0.00	0.00
**COND**	**0.16**	0.00	0.01	**COND**	**0.15**	0.02	0.00	**COND**	0.00	**0.52**	0.05
**HCO3**	0.01	**0.83**	0.01	**HCO3**	0.01	**0.42**	0.45	**HCO3**	**0.17**	0.00	0.00
Cl	0.15	0.04	0.03	Cl	0.14	0.05	0.00	**Cl**	0.04	0.06	**0.59**
**SO4**	0.03	0.07	**0.56**	**SO4-K**	0.02	0.37	**0.52**	SO4-K	0.14	0.00	0.01
Ca	0.13	0.04	0.00	Ca	0.13	0.00	0.01	**Ca**	**0.17**	0.00	0.01
Mg	0.16	0.00	0.02	Mg	0.15	0.00	0.01	Mg	0.15	0.00	0.04
Na	0.15	0.01	0.02	Na	0.15	0.01	0.00	Na	0.16	0.00	0.01
K	0.05	0.01	0.34	K	0.09	0.10	0.00	K	0.00	0.42	0.30
Eigenvalue	6.2	1.1	1.0		6.1	1.3	0.7		5.9	1.4	0.9
% Total variance	69.0	11.9	11.4		68.1	15.0	8.3		66.0	15.7	9.6
Cumulative eigen.	6.2	7.3	8.3		6.1	7.5	8.2		5.9	7.4	8.2
Cumulative%	69.0	80.8	92.2		68.1	83.1	91.3		66.0	81.7	91.3
	PC1 (Env11)	PC2 (Env12)	PC3 (Env13)		PC1 (Env11)	PC2 (Env12)	PC3 (Env13)		PC1 (Env11)	PC2 (Env12)	PC3 (Env13)
**NO3**	0.25	0.21	**0.54**	**NO3**	**0.299**	0.119	**0.324**	**NO3**	**0.36**	0.00	0.20
**NO2**	**0.34**	0.01	0.16	**NO2**	**0.239**	0.263	0.304	**NO2**	0.10	**0.74**	0.01
**NH4**	**0.34**	0.01	0.16	**NH4**	**0.319**	0.129	0.082	NH4	0.28	0.26	0.08
**PO4**	0.06	**0.78**	0.15	**PO4**	0.143	**0.489**	0.289	**PO4**	0.26	0.00	**0.72**
Eigenvalue	2.1	1.0	0.5		2.1	1.1	0.5		1.8	1.0	0.7

% Total variance	53.7	25.5	11.4		52.7	27.1	12.4		46.2	24.9	17.5
Cumulative eigen.	2.1	3.2	3.6		2.1	3.2	3.7		1.8	2.8	3.5
Cumulative%	53.7	79.2	90.6		52.7	79.8	92.2		46.2	71.1	88.6
