# Supplementary material for: Hydrology Affects Environmental and Spatial Structuring of Microalgal Metacommunities in Tropical Pacific Coast Wetlands
Source: PLoS One. 2016 Feb 22;11(2):e0149505. doi: 10.1371/journal.pone.0149505 (PMC4762632; doi:10.1371/journal.pone.0149505)
Supplement: S3 Table — Their life form and body size are indicated. Life form: p-plankton, b-benthic; GALD: greatest axial linear dimension. (PDF) [file pone.0149505.s003.pdf]

**Species**	**life form**	**GALD (µm)**
Actinastrum hantzschii Lagerh. 1882	p	36
Amphikrikos minutissimus Kors. 1953	p	6
Ankistrodesmus gracilis (Reinsch) Kors. 1953	p	32
Ankistrodesmus spiralis (Turn.) Lemm. 1908	p	52
Ankistrodesmus sp.	p	45
Botryococcus braunii Kütz. 1849	p	50
Chlamydomonas sp.	p	15
Chlorella spp.	p	4
Chlorolobion glareosum (Hind.) Kom. 1979	p	6
Closteriopsis acicularis (G.M. Smith) Belch. & Swale 1962	p	102
Closterium aciculare West 1860	b	312
Closterium acutum Bréb. 1848	b	118
Closterium gracile Bréb. 1848	b	60
Closterium kuetzingii Bréb. 1856	b	286
Closterium lineatum Ehrenb. 1848	b	400
Closterium parvulum Näg. 1849	b	115
Coelastrum astroideum De-Not. 1867	p	35
Coelastrum microporum Näg. 1855	p	42
Coelastrum reticulatum (Dang.) Senn 1899	p	41
Coelastrum sp.	p	44
Coenocystis sp.	p	30
Cosmarium pygmaeum Archer 1864	b	11
Cosmarium sp. (pequeño 8*7µm)	b	8
Cosmarium sp. (mediano 18*15 µm)	b	18
Cosmarium sp. (grande 75*60µm)	b	75
Crucigenia tetrapedia (Kirchn.) W. & G.S. West 1902	p	11
Crucigeniella apiculata (Lemm.) Kom. 1974	p	16
Crucigeniella crucifera (Wolle) Kom. 1974	p	20
Crucigeniella pulchra (W. & G.S. West) Kom. 1974	p	21
Crucigeniella rectangularis (Näg.) Kom. 1974	p	18
Desmatractum indutum (Geitl.) Pasch. 1930	p	50
Dictyosphaerium ehrenbergianum Näg. 1849	p	62
Dictyosphaerium pulchellum Wood 1872	p	41
Dictyosphaerium subsolitarium Van Goor 1924	p	15
Dictyosphaerium cf. *tetrachotomum* Printz 1914	p	39
Dictyosphaerium sp.	p	31
Didymocystis comasii Kom. 1983	p	8
Didymocystis inermis (Fott) Fott 1973	p	10
Didymocystis sp.	p	9
Dimorphococcus lunatus A. Br. 1855	p	13
Euastrum cf. *verrucosum* Ehrenb. 1848	b	75
Euastrum sp.	b	30
Eutetramorus sp.	p	15
Franceia sp.	p	6
Golenkinia radiata Chod. 1894	p	35
Golenkiniopsis chlorelloides (Lund) Fott 1981	p	10
Gonatozygon cf. *monotaenium* De Bary 1856	b	215
Gonatozygon sp.	b	180
Granulocystis helenae Hind. 1977	p	10
Granulocystopsis pseudocoronata (Kors.) Hind. 1977	p	12

Kirchneriella contorta (Schmidle) Bohl. 1897	p	31
Kirchneriella irregularis (G.M. Smith) Kors. 1953	p	35
Kirchneriella cf. *subcapitata* Kors. 1953	p	20
Kirchneriella sp. Schmidle	p	29
Lagerheimia balatonica (Scherff. in Kol) Hind. 1978	p	11
Lagerheimia chodati Bern. 1908	p	25
Lagerheimia ciliata (Lagerh.) Chod. 1895	p	40
Micrasterias radiosa Ralfs 1848	p	150
Monomastix sp.	p	15
Monoraphidium arcuatum (Kors.) Hind. 1970	p	40
Monoraphidium circinale (Nyg.) Nyg. 1979	p	10
Monoraphidium contortum (Thur.) Kom.-Legn. 1969	p	25
Monoraphidium komarkovae Nyg. 1979	p	110
Monoraphidium minutum (Näg.) Kom.-Legn. 1969	p	9
Monoraphidium tortile (W. & G.S. West) Kom.-Legn. 1969	p	20
Nephrochlamys subsolitaria (G.S. West) Kors. 1953	p	10
Nephrocytium agardhianum Näg. 1849	p	52
Nephroselmis olivacea Stein 1878	p	9
Oocystis lacustris Chod. 1897	p	8
Oocystis marssonii Lemm. 1898	p	12
Pandorina morum (O.F. Müller) Bory 1824	p	40
Pediastrum simplex Meyen 1829	p	83
Pediastrum tetras (Ehrenb.) Ralfs 1844	p	20
Planctonema lauterbornii Schmidle 1903	p	57
Pleurotaenium sp.	p	450
Pteromonas sp.	p	15
Quadrigula sp.	p	35
Scenedesmus aculeolatus Reinsch 1877	p	20
Scenedesmus acutus Meyen 1829	p	20
Scenedesmus bicaudatus Dedus. 1925	p	30
Scenedesmus ecornis (Ehrenb.) Chod. 1926	p	26
Scenedesmus cf. *granulatus* W.& G.S. West 1897	p	25
Scenedesmus intermedius Chod. 1926	p	25
Scenedesmus obtusus Meyen 1829	p	24
Scenedesmus ovalternus Chod. 1926	p	20
Scenedesmus praetervisus Chod. 1926	p	20
Scenedesmus pseudoarmatus Hortob. 1969	p	25
Scenedesmus quadricauda (Turp.) Bréb. 1913	p	30
Scenedesmus regularis Svir. 1924	p	15
Scenedesmus sempervirens Chod. 1913	p	30
Scenedesmus cf. *semipulcher* Hortob. 1960	p	29
Scenedesmus cf. *sooi* Hortob. 1954	p	15
Scenedesmus verrucosus Roll 1925	p	25
Scenedesmus sp.	p	20
Schroederia sp.	p	30
Sorastum spinulosum Näg. 1849	p	40
Spermatozopsis exsultans Kors. 1913	p	9
Sphaerellopsis sp.	p	15
Sphaerocystis sp.	p	40
Staurastrum sp.	p	30
Staurodesmus sp.	p	15
Tetrachlorella sp.	p	12
Tetraedron caudatum (Corda) Hansg. 1888	p	15

Tetraedron minimum (A. Br.) Hansg. 1888	p	10
Tetraedron triangulare Kors. 1953	p	12
Tetraselmis sp.	p	12
Tetrastrum heteracanthum (Nordst.) Chod. 1895	p	30
Tetrastrum punctatum (Schmidle) Ahlstr. & Tiff. 1934	p	10
Tetrastrum triangulare (Chod.) Kom. 1974	p	11
Treubaria triappendiculata Bern. 1908	p	29
Treubaria quadrispina (G.M. Smith) Fott & Kovac 1975	p	20
Treubaria schmidlei (Schröd.) Fott & Kovac 1975	p	30
Xanthidium sp.	p	42
Indeterminate cocal	p	11
Chromulina sp.	p	6
Chrysococcus sp.	p	8
Desmarella sp.	p	8
Kephyrion sp.	p	10
Mallomonas sp.	p	15
Ochromonas sp.	p	10
Centritractus belenophorus Lemm. 1900	p	60
Centritractus sp.	p	30
Gonichloris sp.1	p	15
Gonichloris sp.2	p	18
Chroomonas sp.	p	12
Cryptomonas erosa Ehrenb. 1932	p	25
Cryptomonas marssonii Skuja 1948	p	30
Cryptomonas cf. *ovata* Ehrenb. 1932	p	20
Cryptomonas phaseolus Skuja 1948	p	12
Cryptomonas rostratiformis Skuja 1956	p	40
Cryptomonas sp.	p	35
*Plagioselmis nannoplanctica (*Skuja) Nov. 1994	p	12
Gymnodinium sp.	p	20
Katodinium sp.	p	12
Peridiniopsis sp.	p	30
Peridinium umbonatum Stein 1883	p	31
Peridinium willei Huitfeld-Kaas 1900	p	65
Peridinium sp.	p	25
Cyste	p	20
Cryptoglena sp.	p	12
Euglena acus Ehrenb. 1830	p	120
Euglena ehrenbergii Klebs 1883	p	220
Euglena gaumei All. & Lef. 1930	p	65
Euglena cf. *gymnodinioides* Zakrys 1986	p	30
Euglena oxyuris Schmarda 1846	p	240
Euglena spirogyra Ehrenb. 1838	p	90
Euglena sp. (curvada 18x10µm)	p	18
Euglena spp.	p	25
Lepocinclis fusiformis (Carter) Lemm. emend. Conr. 1901	p	30
Lepocinclis ovum (Ehrenb.) Lemm. 1910	p	20
Lepocinclis texta (Duj.) Lemm. emend. Conr. 1934	p	45
Phacus inflexus (Kiss.) Pochm. 1942	p	25
Phacus curvicauda Swir. 1915	p	30
Phacus longicauda (Ehrenb.) Duj. 1841	p	110
Phacus orbicularis Hübn. 1886	p	60
Phacus parvulus Klebs 1883	p	25

Phacus polytrophos Pochm. 1942	p	21
Phacus pyrum (Ehrenb.) Stein 1878	p	35
Phacus suecicus Lemm. 1913	p	25
Phacus sp.	p	17
Strombomonas fluviatilis (Lemm.) Defl. 1930	p	34
Strombomonas cf. *maxima* (Skv.) Defl. 1930	p	55
Strombomonas verrucosa (Daday) Defl. 1930	p	25
Strombomonas spp.	p	30
Trachelomonas armata (Ehrenb.) Stein 1878	p	50
Trachelomonas globularis (Awer.) Lemm. 1910	p	15
Trachelomonas hispida (Perty) Stein emend. Defl. 1926	p	30
Trachelomonas cf. *pulcherrima* Playf. 1915	p	20
Trachelomonas robusta Swir. emend. Defl. 1926	p	30
Trachelomonas cf. *verrucosa* Stokes 1887	p	15
Trachelomonas volvocina Ehrenb. 1838	p	10
Trachelomonas volvocinopsis Swir. 1914	p	16
Trachelomonas spp.	p	20
Acanthoceras zachariasii (Brun) Simonsen 1979	b	52
Achnanthes catenata Bily & Marvan 1959	b	14
Achnanthes hungarica (Grunow) Grunow 1880	b	30
Achnanthes lanceolata (Bréb.) Grunow 1880	b	15
Achnanthes sp.	b	15
Amphipleura pellucida (Kütz.) Kütz. 1844	b	103
Amphora sp.	b	45
Aulacoseira granulata (Ehrenb.) Simonsen 1979	p	80
Aulacoseira granulata var. *angustissima* (O.Müller) Simonsen 1979	p	110
Aulacoseira sp.	p	108
Bacillaria paxillefera (O.Müller) Hendey 1951	b	105
Caloneis sp.	b	80
Cocconeis placentula Ehrenb. 1838	b	45
Craticula ambigua (Ehrenb.) Mann 1990	b	60
Cyclotella meneghiniana Kütz. 1844	p	15
Cyclotella ocellata Pantocsek 1901	p	12
Cyclotella pseudostelligera Hustedt 1939	p	10
Cyclotella sp.	p	8
Cymbella minuta Hilse 1862	b	20
Cymbella sp.	b	25
Diadesmis confervacea Kütz. 1844	b	20
Diploneis cf. *finnica* (Ehrenb.) Cleve 1891	b	61
Diploneis interrupta (Kütz.) Cleve 1894	b	49
Diploneis oblongella (Naeg.) Cleve-Euler 1922	b	20
Diploneis sp.	b	25
Entomoneis alata (Ehrenb.) Ehrenb. 1845	b	80
Eunotia bilunaris (Ehrenb.) Mills 1934	b	80
Eunotia cf. *monodon* (Ehrenb.) 1843	b	120
Eunotia sp.	b	70
Fallacia pygmaea (Kütz.) Stickle & Mann 1990	b	40
Fragilaria biceps (Kütz.) Lange-Bertalot 1991	b	304
Fragilaria crotonensis Kitton 1869	b	80
Fragilaria ulna var. *acus (*Kütz.) Lange-Bertalot 1980	b	150
Fragilaria sp.	b	81
Gomphonema augur Ehrenb. 1840	b	40

Gomphonema gracile Ehrenb. 1838	b	60
Gomphonema parvulum (Kütz.) Kütz. 1849	b	25
Gomphonema sp.	b	25
Gyrosigma acuminatum (Kütz.) Rabenhorst 1853	b	110
Hantzschia amphioxys (Ehrenb.) Grunow 1880	b	30
Melosira varians Agardh 1827	p	102
Navicula cryptocephala Kütz. 1844	p	30
Navicula cf. *leptostriata* Jorg. 1948	p	35
Navicula cf. *pusio* Cleve 1895	p	15
Navicula radiosa Kütz. 1844	p	80
Navicula subrhynchocephala Hustedt 1935	p	39
Navicula veneta Kütz. 1844	p	20
Navicula spp.	p	60
Nitzschia acicularis (Kütz.) W.Smith 1853	b	83
Nitzschia amphibia Grunow 1862	b	35
Nitzschia cf. *clausii* Hantzs. 1860	b	30
Nitzschia closterium (Ehrenb.) W.Smith 1853	b	197
Nitzschia denticula Grunow 1880	b	35
Nitzschia dissipata (Kütz.) Grunow 1862	b	45
Nitzschia gracilis Hantzs. 1860	b	65
Nitzschia palea (Kütz.) W.Smith 1856	b	41
Nitzschia filiformis (W.Smith) Van Heurck 1896	b	60
Nitzschia microcephala Grunow 1878	b	15
Nitzschia obtusa W.Smith 1853	b	235
Nitzschia reversa W.Smith 1853	b	135
Nitzschia sigma (Kütz.) W.Smith 1853	b	45
Nitzschia sigmoidea (Nitzs.) W.Smith 1853	b	325
Nitzschia spp.	b	40
Pinnularia acrosphaerica W.Smith 1853	b	92
Pinnularia subcapitata Gregoy 1856	b	45
Pinnularia viridis (Nitzs.) Ehrenb. 1843	b	140
Pinnularia sp.	b	90
Pleurosigma sp.	b	120
Rhizosolenia longiseta Zacharias 1893	b	100
Rhopalodia gibba (Ehrenb.) O.Müller 1895	b	164
Rhopalodia gibberula (Ehrenb.) O.Müller 1895	b	60
Sellaphora pupula (Kütz.) Meresch. 1902	b	50
Stauroneis phoenicenteron (Nitzs.) Ehrenb. 1843	b	145
Stauroneis sp.	b	120
Synedra ulna	b	310
Tryblionella sp.	b	60
Anabaena spp.	p	150
Anabaenopsis elenkinii Miller 1923	p	63
Aphanizomenon sp.	p	97
Aphanocapsa elachista W. & G.S. West 1894	p	21
Aphanocapsa incerta (Lemm.) Cronb. & Kom. 1994	p	40
Aphanocapsa sp.	p	44
Aphanothece cf. *clathrata* W. & G.S. West 1906	p	78
Aphanothece stagnina (Sprengel) Braun 1863	p	30
Aphanothece sp.	p	29
Chroococcus minutus (Kütz.) Näg. 1849	p	5
Chroococcus cf. *turgidus* (Kütz.) Näg. 1849	p	11

Chroococcus sp.	p	10
Cylindrospermum sp.	p	52
Geitlerinema acutissimum (Kuff.) Anag. 1989	b	202
Geitlerinema amphibium (Agardh. ex Gomont) Anag. 1989	p	150
Geitlerinema sp.	b	100
Glaucospira sp.	b	20
Jaaginema geminatum (Menegh. ex Gomont) Anag. & Kom. 1988	b	150
Jaaginema sp.	b	101
Lyngbya sp.	b	306
Merismopedia marssonii Lemm. 1900	p	15
Merismopedia punctata Meyen 1839	p	20
Merismopedia tennuissima Lemm. 1898	p	32
Microcystis sp.	p	20
Oscillatoria ornata Kütz. ex Gomont 1892	b	99
Oscillatoria subbrevis Schmidle 1901	b	398
Oscillatoria cf. *tenuis* Agardh. ex Gomont 1892	b	504
Oscillatoria sp.	b	500
Pannus sp.	p	61
Phormidium sp.	b	305
Planktolyngbya sp.	p	99
Planktothrix sp.	p	120
Pseudanabaena amphigranulata (Van Goor) Anag. 2001	b	80
Pseudanabaena cf. *catenata* Lauterb. 1915	b	72
Pseudanabaena cf. papillaterminata (Kiselev) Kukk 1959	b	80
Pseudanabaena sp.	b	62
Spirulina spp.	p	97
Synechococcus sp.	p	6
Synechocystis aquatilis Sauvageau 1892	p	8
Woronichinia spp.	p	40
Croococal indeterminada	p	15
Oscillatoriales- sp1	b	40
Oscillatoriales- sp2	b	101
Oscillatoriales- sp3	b	100
